# Reasons for the Reporting Behavior of Japanese Collegiate Rugby Union Players Regarding Suspected Concussion Symptoms: A Propensity Analysis

**DOI:** 10.3390/ijerph20032569

**Published:** 2023-01-31

**Authors:** Keita Suzuki, Satoshi Nagai, Satoru Nishida, Koichi Iwai, Masahiro Takemura

**Affiliations:** 1 Faculty of Health and Sports, Nagoya Gakuin University, 1350 Kamishinano, Seto, Aichi 480-1298, Japan; 2 Faculty of Health Sciences, Tsukuba International University, 6-8-33 Manabe, Tsuchiura, Ibaraki 300-0051, Japan; 3 Faculty of Sport and Health Sciences, Ryutsu Keizai University, 120 Hirahata, Ryugasaki, Ibaraki 301-8555, Japan; 4 Center for Humanities and Sciences, Ibaraki Prefectural University of Health Sciences, 4669-2 Ami, Ami-machi, Inashiki-gun, Ibaraki 300-0394, Japan; 5 Faculty of Health and Sport Sciences, University of Tsukuba, 1-1-1 Tennodai, Tsukuba, Ibaraki 305-8574, Japan

**Keywords:** concussion, reporting behavior, propensity score

## Abstract

While previous research has identified the reasons for the concussion-reporting behavior of rugby union players, the influence of confounding factors such as concussion experience, education, and knowledge of concussion symptoms, any of which may have influenced the results, has not been considered. This study aimed to clarify the reasons for the reporting behavior of college rugby union players regarding suspected concussion symptoms by adjusting for confounding factors using the propensity score. A questionnaire about both concussion knowledge and concussion-reporting behavior was administered to 240 collegiate rugby union players. Of the 208 (86.7%) valid respondents to the questionnaire, 196 (94.2%) had experienced any one symptom of a suspected concussion, such as headache, at least once, and 137 (65.9%) reported symptoms to someone else. This study’s results revealed two important reasons for reporting symptoms: (1) the willingness of players to report experienced symptoms to someone else, along with realizing a concussion, and (2) the willingness of players to report suspected concussion symptoms, despite the absence of a doctor or trainer. These results suggest that providing educational opportunities to recognize suspected concussion symptoms and establishing a team culture of reporting physical problems to someone else is important for improving concussion-reporting behavior.

## 1. Introduction

Systematic reviews have reported that rugby union (RU) has a higher incidence of concussion than other contact/collision sports [1,2]. For a concussion to be collected as an injury record, that is, for the medical staff to check it and adapt a graduated return-to-play protocol, it is necessary for the athletes to report concussion symptoms themselves or for others to detect the symptoms. Therefore, some previous studies have focused on whether concussions have been experienced or unreported in RU players. In RU, 25.0–69.0% of players have experienced a concussion [3,4,5,6,7,8,9,10,11,12,13], of which 46.6–52.5% do not report suspected concussion symptoms [5,12]. About half of RU players will continue playing with concussion symptoms.

RU players do not report concussions for the following three reasons [3,4,5,12]: (1) they do not think the injury is serious; (2) they do not realize it is a concussion; and (3) they do not want to be removed from play. In previous studies of athletes in other sports apart from rugby, the athletes did not report concussions for the same reasons [14,15,16]. The study by Wallace et al. on US high school students also found that respondents were trying to get a college scholarship [17,18]. Moreover, internal factors, such as an individual’s personality and attitudes [19], and external factors, such as the environment and relationships in the surroundings [20], have been reported to influence concussion-reporting behavior. Previous studies have reported that some RU players believe they will continue to play if it is an important game, even if they are experiencing concussion symptoms [6,8,11,13]. Therefore, it is assumed that an athlete’s desire to continue playing is stronger than the impact of the concussion. Suspected concussion symptoms can be divided into two categories: those others can detect and those the individual needs to recognize. Therefore, players must know the concussion symptoms, especially those they need to recognize. However, there is no consensus on the influence of knowing about concussions on reporting behavior [21,22]. While previous studies have identified reasons for not reporting symptoms, it is expected that examining the reasons for reporting will improve reporting behavior.

The results of previous studies may have been influenced by confounding factors such as concussion experience, education, and knowledge of concussion symptoms. A history of concussions is expected to provide experiences of returning to competition after a concussion and the opportunity to obtain more information about concussions from the medical staff. Such experiences positively affect players’ reporting behavior; however, it cannot be denied that they can also have a negative effect. Previous research has reported that lifetime concussion history negatively correlates with athlete self-report accuracy, and recall worsens as the number of lifetime concussions increases [23]. Previous concussions may influence the behavior of players when concussions reoccur. Therefore, we focused on the propensity score (PS), which has recently been used to adjust for confounding factors in observational studies [24,25]. By calculating the PS and incorporating it into the model of the analysis as a covariate, it is possible to adjust for confounding factors such as the number of previous concussions and whether or not the individual has received concussion education; we believe that the factors that genuinely influence concussion-reporting behavior can be identified.

This study’s aims were as follows: (1) to determine the reasons for the behavior of reporting suspected concussion symptoms using PS and adjusting for confounding factors; (2) to clarify knowledge and reporting behavior of Japanese collegiate RU players regarding concussions.

## 2. Materials and Methods

### 2.1. Research Design and Subjects

This study was a retrospective cross-sectional study of Japanese male collegiate RU players. A total of 240 collegiate RU players from five universities participated in the study. The survey was conducted between August 2021 and January 2022. The Ethics Committee of the Faculty of Health and Sport Science at the University of Tsukuba approved the study (Reference number: 021-54). Participants were given an informed consent form to review and indicated consent after completing the questionnaire.

### 2.2. Measures

The questionnaires regarding concussion knowledge, the experience of suspected concussion symptoms, and subsequent behavior were administered to participants using (1) Google Forms or (2) paper and pencil, depending on the resources available in each data-collection situation. The survey lasted 10–15 min based on previous studies [3,4,5,14]. The questionnaire consisted of the following four sections (Appendix A): (1) player profile (age, academic year, and years in RU); (2) knowledge of suspected concussion symptoms; (3) concussion education; and (4) experience of suspected concussion symptoms and subsequent behavior.

The player profile, that is, demographic information collected for this study, was age, academic year, and years of RU experience. Sex was not checked in this study, because only male RU players participated.

In the section on knowledge of suspected concussion symptoms (see Appendix A Q2 and Table 1), we included 33 symptoms; 22 were correct symptoms from Sports Concussion Assessment Tool 5 (SCAT 5) [26], and 11 were incorrect distracters [14]. We calculated the Symptoms Recognized Scores (SRS) as the number of symptoms a player recognized as suspected concussion symptoms out of the 33 symptoms. A higher SRS indicated a better knowledge of suspected concussion symptoms. 

In the section on concussion education, first, participants answered the following question: “have you ever been educated on the symptoms of a suspected concussion, what to do after a concussion occurs, and procedures for returning to play?” Next, we asked when they received the education above (elementary, junior high, high school, or college) and the education form, such as school classes or instruction from a trainer.

Experience of suspected concussion symptoms was defined as “when a player had experienced at least one of the 22 suspected concussion symptoms from SCAT 5 [26,27] after a blow to the head, face, neck, or other parts of the body with an impulsive force transmitted to the head”. Furthermore, it did not matter whether they reported those symptoms to anyone, or whether a doctor assessed them. Participants responded to the total number of experiences they have had of these symptoms and how many times they had experienced them in elementary, junior high, high school, and college. In addition, participants selected the timing of the most recent onset of symptoms they experienced. We asked if they had seen a doctor after experiencing symptoms, in what situations they experienced the symptoms (for example, games or practices), whether others around them detected the symptoms, whether they reported the symptoms to anyone, and if so, to whom they reported their symptoms.

Finally, participants responded as to why they reported or did not report suspected concussion symptoms. According to previous studies, the reasons for reporting symptoms were based on the reasons for not reporting [3,4,5,14]. The reasons for reporting behavior were rated on a 5-point Likert scale ranging from 1 (*strongly disagree* or *strongly agree*) to 5 (*strongly agree* or *strongly disagree*).

We transformed the reasons for reporting suspected concussion symptoms as follows: (1) *strongly disagree* and *disagree* were both categorized as negative, and (2) *strongly agree* and *agree* were both categorized as positive. In contrast, the reasons for not reporting suspected concussion symptoms were transformed as follows: (1) *strongly agree* and *agree* were both categorized as negative, and (2) *strongly disagree* and *disagree* were both categorized as positive. Reasons for reporting behavior were created using questions inverted and used as a single question in the statistical analysis.

### 2.3. Statistical Analysis

Descriptive data (means, 95% confidence interval (CI), median, interquartile range, frequencies, and percentages) are presented where appropriate. The numerical data were checked for normality using the Shapiro–Wilk normality test. Due to the non-normality of the numerical data, Mann–Whitney U tests were used to compare the SRS of players who received concussion education and those who did not, players who reported suspected concussion symptoms and those who did not, and players with a concussion and those without a concussion. Effect sizes (*r*) were calculated to determine the magnitude of the difference in SRS. Effect sizes were calculated by dividing the Z-scores by the square root of the sample size [28]. The effect sizes used were: 0.1 (small effect), 0.3 (moderate effect), and ≥0.5 (large effect) [29]. The chi-square tests (Pearson’s chi-square or Fisher’s exact test, as appropriate) were used to compare differences in reporting suspected concussion symptoms (yes vs. no) for categorical data. Pearson’s chi-square test was also used to assess differences based on players with concussions and those without for each recognition of suspected concussion symptoms.

A generalized linear model (GLM) was used to determine the reasons for concussion-reporting behavior. Initially, a PS was calculated for the influence of the following factors on concussion-reporting behavior: years of playing rugby, SRS, concussion education, and the number of previous concussions. Subsequently, the ROC (Receiver Operating Characteristic) analysis was performed to confirm the accuracy of the classification of concussion-reporting behavior based on PS, and concordance statistics were calculated.

Next, the inverse probability weighting (IPW) treatment was adjusted for all participants based on PS. We calculated IPW as follows [25]: 1/PS for players who reported concussion symptoms; and 1/(1–PS) for those who did not report them.

Finally, the GLM was performed using IPW as a covariate. The dependent variable was “whether the player reported symptoms or not”, and the independent variable was “reasons for reporting behavior”. For the independent variables, we selected responses of “neither” as the reference category. We calculated the odds ratio (OR) and 95% CI using GLM. A certain reason with an OR > 1.0 is more likely to lead to concussion reporting, whereas a certain reason with an OR < 1.0 is less likely to lead to concussion reporting. The Wald χ2 value, calculated by dividing the regression coefficient (B) by the standard error (SE) and squaring, indicates the impact of each reason on concussion-reporting behavior. In addition, an unadjusted GLM was performed to confirm the influence on the results of adjusting the IPW. All statistical analyses were performed using the SPSS version 28.0 package (IBM Japan Inc., Tokyo, Japan). Statistical significance was set at *p* < 0.05.

## 3. Results

### 3.1. Player Profile

There were 208 valid responses (86.7%). The mean age was 20.3 years (95% CI, 20.1–20.5), and the mean number of years playing in RU was 9.1 years (95% CI, 8.6–9.7). The players’ college grades were evenly distributed (Table S1).

### 3.2. Knowledge of Concussion Symptoms

The SRS was 22.7 (95% CI, 22.2–23.3), with an overall recognition rate of 68.8%. Players accurately identified (>90%) the following five concussion symptoms: “balance problems”, “dizziness”, “nausea or vomiting”, “headache”, and “skin rash” (Table 1). Conversely, the recognition rate of symptoms associated with emotional changes, such as “more emotional”, “irritability”, and “sadness”, was <30%. Players who had not experienced a concussion were significantly more likely to correctly recognize the “sensitivity to light” than those who had experienced a concussion (91.7% vs. 48.5%; *p* = 0.005).

When comparing SRS between players with and without concussion education, players with concussion education had a significantly higher SRS than those without concussion education (23.3 vs. 21.9; *p* = 0.005; *r* = 0.20, small effect). When comparing SRS between players who reported concussion symptoms and those who did not, players who reported a concussion had a higher SRS than those who did not report a concussion; however, the difference was not statistically significant (22.8 vs. 22.3; *p* = 0.440, *r* = 0.06, trivial effect). When comparing SRS between players who experienced concussion symptoms and those who did not, players who did not experience concussion symptoms had a higher SRS than those who experienced concussion symptoms; however, the difference was not statistically significant (23.5 vs. 22.7; *p* = 0.535, *r* = 0.04, trivial effect).

### 3.3. Concussion Education

One hundred twenty-one players (58.2%) received concussion education. However, most players (*n* = 94) received concussion education in high school (Table S2). Furthermore, 87 and 60 players received concussions from club advisors and trainers, respectively (Table S2).

### 3.4. Experience of Suspected Concussion Symptoms and Subsequent Behavior

Of the 208 respondents, 196 (94.2%) had experienced suspected concussion symptoms at least once. In addition, many RU players experienced the following three suspected concussion symptoms: “headache” (84.2%), “balance problems” (74.0%), and “dizziness” (73.5%) (Table 2).

The mean number of times RU players experienced suspected concussion symptoms was 2.8 (95% CI, 2.5–3.1). The highest mean number of times RU players experienced suspected concussion symptoms was 1.6 (95% CI, 1.3–1.8) in high school (Table S3).

The latest suspected concussion symptoms occurred most often immediately after a head impact (35.2%) or after a game or practice (29.6%). In addition, some players (8.7%) experienced symptoms when they woke up the following day (Table 3).

Sixty players (28.8%) received medical attention from a doctor after experiencing suspected concussion symptoms. In addition, 145 players (69.7%) experienced suspected concussion symptoms in games (Table S4).

One hundred twenty-five (60.1%) players had their suspected concussion symptoms detected by someone else, and 137 (65.9%) reported them to someone else themselves (Table S4). In addition, 104 and 77 players reported concussion symptoms to club advisors/coaches and trainers, respectively (Table S4).

The frequency of reporting symptoms differed depending on whether or not the players had received concussion education; however, the difference was not statistically significant (*p* = 0.355; Table 4). Conversely, the frequency of reporting symptoms differed significantly depending on whether or not others detected the symptoms (*p* < 0.001; Table 4).

Based on the PS, the discrimination rate for predicting whether players reported suspected concussion symptoms was 71.8%, with concordance statistics of 0.59. Table 5 shows the reasons for concussion-reporting behavior, using the IPW as a covariate. GLM results showed that the following three reasons strongly influenced concussion-reporting behavior (Wald χ^2^ = 15.71, 15.68, and 10.46, respectively): (1) players thought that realizing it was an injury/symptom that they should report influenced their reporting behavior (OR = 14.25 [95% CI, 3.83–53.03]; *p* < 0.001); (2) players thought that not realizing that they had a suspected concussion but thought it was a symptom that they should report influenced their reporting behavior (OR = 26.30 [95% CI, 5.21–132.67]; *p* < 0.001); and (3) players thought that the absence of a doctor or trainer had little effect on reporting behavior (OR = 26.47 [95% CI, 3.64–192.72]; *p* = 0.001).

The following five reasons were found to lead to reporting suspected concussion symptoms (Table 5): (1) players wanted to continue with the game/practice that day but thought it was a symptom that they should report; (2) players did not think reporting the symptoms might ruin the atmosphere of the team at games/practices; (3) players recognized suspected concussion symptoms; (4) players thought that the presence of a doctor or trainer had little effect on reporting behavior; and (5) players thought that leaving the game/practice would not affect their future careers. Furthermore, the following four reasons were found to lead to nondisclosure of suspected concussion symptoms (Table 5): (1) players did not realize at the time that they had a concussion; (2) players wanted to continue with the game/practice that day; (3) players thought others would think less of them if they reported their symptoms; and (4) players wanted to continue with the game/practice the next day.

## 4. Discussion

Previous studies have identified the reasons for concussion-reporting behavior in RU players; however, the influence of confounding factors such as concussion experience, education, and knowledge of concussion symptoms, which may have influenced the results, were not considered. Therefore, this study aimed to clarify the reasons for the reporting behavior of college RU players regarding suspected concussion symptoms by adjusting for confounding factors using PS. In addition, this study clarified intriguing reasons not identified in previous studies by adjusting for PS.

### 4.1. Knowledge and Education on Concussion

The symptom recognition rate of concussion was 68.8% among Japanese college RU players; this result was higher than that in previous studies of Japanese collegiate athletes (52.5%), including RU players [30]. This suggests that RU players in Japan have a better understanding of concussions than other sports players, because they have more opportunities to experience concussions and closely observe their teammates experiencing them. However, compared with studies of RU players in other countries apart from Japan, the symptom recognition rates in the present study were similar [7,31] or approximately 20% lower [32]. In addition, previous studies have reported that concussion education increases knowledge of concussions [33]. Therefore, it is important to provide opportunities for concussion education to players to promote their understanding of concussion symptoms.

Approximately 60% of Japanese college RU players in this study had received concussion education; this result was similar to [11] or slightly higher than [5,7] in previous studies. In addition, club advisors provided concussion education most frequently (Table S2), which is consistent with previous studies [4,13]. Concussion education is necessary for understanding symptoms and reporting behavior; however, the quality of concussion education in Japan needs to be higher. This can be discussed in conjunction with this study’s symptom recognition rate results. In Japan, concussion education is optional for club advisors, and it is unclear to what degree they understand the issue. Previous studies have examined concussion knowledge among coaching staff [32,34,35]. The results showed that the coaching staff has high concussion knowledge. However, there are no similar surveys in Japan. Moreover, based on this study’s results and previous findings, RU players often report suspected concussion symptoms to the coaching staff [3,4,13,36]. Therefore, it is important to provide concussion education not only to players but also to the coaching staff, such as club advisors.

### 4.2. Experience of Suspected Concussion Symptoms

Of the 208 respondents, 196 (94.2%) had experienced suspected concussion symptoms at least once, which was notably higher than in previous studies [3,4,5,6,7,8,9,10,11,12,13]. In addition, 137 of the 196 players (69.9%) reported concussion symptoms to others, a higher reporting rate than in previous studies [5,12]. These results suggest that Japanese RU players are likely to experience and report suspected concussion symptoms. However, the fact that approximately 30% of players do not report symptoms and almost all players have experienced concussion symptoms at least once is a serious issue for player welfare. We believe that developing rugby-specific educational awareness programs that include technical interventions, such as the importance of approaches to prevent tackling with inappropriate head placement, which is a frequent cause of concussion [37], will be necessary to solve the problem.

### 4.3. Reasons Leading to Reports of Concussions

Eight reasons led to the behavior of reporting suspected concussion symptoms, while four led to nondisclosure. Notably, some results were in line with previous studies [3,4,5,12]: (1) whether players realized a concussion; and (2) their intention to continue playing. Furthermore, unlike previous studies [9,34], Japanese college RU players did not feel pressure from others to report symptoms. In addition, players thought that leaving the game/practice would not affect their future careers if they reported their symptoms; this result contrasts with that of a previous study [17]. Players did not think reporting the symptoms might ruin the atmosphere of the team, whereas they thought others would think less of them if they reported their symptoms (Table 5).

The most important finding was that players thought they should report the symptoms experienced, regardless of whether they realized it was a concussion. This suggests that Japanese RU players may have formed the habit of reporting to someone when they feel any physical problem. Furthermore, this study found that nondisclosure of symptoms was influenced by the perception that others would think less of them for reporting symptoms. In student sports, they often worry about how their teammates will perceive them for reporting concussion symptoms [38]. Therefore, it is necessary for everyone on the team to give psychological consideration to athletes to make it easier for them to report physical problems, not just concussions.

Interestingly, the absence of a doctor or trainer did not influence the disclosure of suspected concussion symptoms. This was a new finding based on the adjustment for confounding factors using PS. In addition, this result supports the above discussion that Japanese RU players habitually report to someone when they experience physical problems. Educating players to report any physical problems, including concussion symptoms, to someone is important. However, the medical staff has a vital role in player welfare, including assessing concussions and subsequent management of the return to play. Therefore, a system for medical staff to support RU players must be established.

Whether or not the players realized that they were experiencing concussion symptoms affected their reporting behavior. To date, there has yet to be a consensus that concussion education leads to reporting behavior. In this study, players who received concussion education were more likely to report symptoms than those who did not; however, the difference was not statistically significant (*p* = 0.355; Table 4). However, the GLM results suggest that whether players realize they have a concussion can affect their disclosure of symptoms. Therefore, it is necessary to continue to educate players to recognize and understand concussion symptoms. Moreover, we believe it is essential for player welfare to establish a team culture of reporting suspected concussion symptoms to someone when realized.

### 4.4. Limitations

This study generated important information; however, several limitations should be addressed. First, the experience of suspected concussion symptoms and the subsequent behavior was asked in response to the last time the players experienced a concussion. Therefore, recall bias might have affected this study’s results. Second, we did not ask whether medical staff, such as doctors or trainers, were present when players experienced the suspected concussion symptoms. Medical staff affiliations are not mandatory in Japanese college RU. However, this study’s results suggest that providing educational opportunities to recognize suspected concussion symptoms and establishing a team culture of reporting physical problems to someone are important for player welfare and improving concussion-reporting behavior. Therefore, we believe that medical staff is essential in providing educational opportunities and establishing a team culture of reporting symptoms. Future research needs to include a question about the affiliation of full-time medical staff.

## 5. Conclusions

To the best of our knowledge, this is the first study to clarify the reasons for the reporting behavior of college RU players regarding suspected concussion symptoms by adjusting for confounding factors using PS. As a result, this study clarified intriguing reasons not identified in previous studies by adjusting for PS. This study’s results revealed two essential reasons: (1) the willingness of players to report symptoms that occur to them to someone else, in addition to when realizing they are suffering from a concussion; and (2) the willingness of players to report suspected concussion symptoms, despite the absence of a doctor or trainer. Our results suggest that it is necessary to improve concussion education for those involved in rugby, such as players and coaches. The development of rugby-specific educational awareness programs that include technical interventions is expected. In addition, the medical staff has a vital role in player welfare, including assessing concussions, subsequent management of the return to play, and concussion education. Therefore, a system for medical staff to support RU players must be established. However, due to financial problems and the number of trainers, not all teams may have immediate access to medical staff support. Therefore, we believe establishing a team culture of reporting physical problems to someone is important for player welfare and improving concussion-reporting behavior.

## Figures and Tables

**Table 1:** Recognition of suspected concussion symptoms and total scores.

	All Respondents (N = 208)		Respondents with Concussion (N = 196)		Respondents without Concussion (N = 12)	
Knowledge item ^1^	n	(%)	n	(%)	n	(%)
Balance problems (true)	199	(95.7)	187	(95.4)	12	(100.0)
Dizziness (true)	197	(94.7)	186	(94.9)	11	(91.7)
Nausea or vomiting (true)	191	(91.8)	181	(92.3)	10	(83.3)
Headache (true)	190	(91.3)	180	(91.8)	10	(83.3)
Skin rash (false)	189	(90.9)	179	(91.3)	10	(83.3)
Blurred vision (true)	187	(89.9)	176	(89.8)	11	(91.7)
Bleeding from the mouth (false)	184	(88.5)	174	(88.8)	10	(83.3)
“Do not feel right” (true)	180	(86.5)	170	(86.7)	10	(83.3)
Abnormal sense of smell (false)	174	(83.7)	166	(84.7)	8	(66.7)
Abnormal sense of taste (false)	171	(82.2)	163	(83.2)	8	(66.7)
“Pressure in head” (true)	169	(81.3)	158	(80.6)	11	(91.7)
Feeling slowed down (true)	169	(81.3)	157	(80.1)	12	(100.0)
Bleeding from the ears (false)	168	(80.8)	159	(81.1)	9	(75.0)
Difficulty concentrating (true)	168	(80.8)	157	(80.1)	11	(91.7)
Confusion (true)	165	(79.3)	154	(78.6)	11	(91.7)
Difficulty remembering (true)	163	(78.4)	152	(77.6)	11	(91.7)
Joint stiffness (false)	159	(76.4)	151	(77.0)	8	(66.7)
Nosebleed (false)	151	(72.6)	143	(73.0)	8	(66.7)
Numbness in arms (false)	147	(70.7)	141	(71.9)	6	(50.0)
Black eye (false)	142	(68.3)	134	(68.4)	8	(66.7)
Fever (false)	141	(67.8)	132	(67.3)	9	(75.0)
Fatigue or low energy (true)	139	(66.8)	130	(66.3)	9	(75.0)
Feeling like “in a fog” (true)	122	(58.7)	113	(57.7)	9	(75.0)
Neck muscle weakness (false)	110	(52.9)	107	(54.6)	3	(25.0)
Sensitivity to light (true) ^2^	106	(51.0)	95	(48.5)	11	(91.7)
Trouble falling asleep (true)	103	(49.5)	97	(49.5)	6	(50.0)
Neck pain (true)	101	(48.6)	94	(48.0)	7	(58.3)
Drowsiness (true)	99	(47.6)	93	(47.4)	6	(50.0)
Nervous or anxious (true)	99	(47.6)	91	(46.4)	8	(66.7)
Sensitivity of noise (true)	74	(35.6)	68	(34.7)	6	(50.0)
More emotional (true)	61	(29.3)	56	(28.6)	5	(41.7)
Irritability (true)	61	(29.3)	57	(29.1)	4	(33.3)
Sadness (true)	47	(22.6)	43	(21.9)	4	(33.3)
Total score	22.7	(22.2–23.3)	22.7	(22.1–23.2)	23.5	(21.7–25.3)

^1^ The correct response for each item is indicated in parentheses. ^2^ *p* < 0.05 (Pearson’s chi-square test) Categorical data are presented as frequency and percentage (%). Continuous data are presented as mean and 95% CI.

**Table 2:** Experiences of suspected concussion symptoms in collegiate rugby union players.

Symptoms	Frequency	(%) ^1^
Headache	165	(84.2)
Balance problems	145	(74.0)
Dizziness	144	(73.5)
“Do not feel right”	113	(57.7)
Blurred vision	104	(53.1)
Neck pain	96	(49.0)
Feeling slowed down	89	(45.4)
Nausea or vomiting	88	(44.9)
“Pressure in head”	82	(41.8)
Difficulty concentrating	82	(41.8)
Confusion	71	(36.2)
Fatigue or low energy	69	(35.2)
Difficulty remembering	58	(29.6)
Nervous or anxious	45	(23.0)
Drowsiness	44	(22.4)
Sadness	39	(19.9)
Feeling like “in a fog”	37	(18.9)
Trouble falling asleep	28	(14.3)
More emotional	25	(12.8)
Sensitivity to light	23	(11.7)
Irritability	21	(10.7)
Sensitivity to noise	15	(7.7)

^1^ N = 196.

**Table 3:** Timing of the last onset of suspected concussion symptoms.

Questions	Frequency	(%) ^1^
When was the latest symptom you experienced so far?		
Immediately after hitting your head	69	(35.2)
After practice/games	58	(29.6)
On the way home	29	(14.8)
From arriving home to having a meal	7	(3.6)
When taking a bath	4	(2.0)
From leaving the bath to going to bed	5	(2.6)
In the middle of sleeping	1	(0.5)
When waking up the next morning	18	(9.2)
After that	5	(2.6)

^1^ N = 196.

**Table 4:** The impact of concussion education and symptom detection on reporting behavior.

	Reporting Concussion Symptoms					
	Yes		No			
	Frequency	(%) ^1^	Frequency	(%) ^1^	Cramer’s V	*p*
Concussion education					0.066	0.355
Yes	84	(61.3)	32	(38.7)		
No	53	(54.2)	27	(45.8)		
Detecting concussion symptoms					0.477	<0.001
Yes	108	(78.8)	17	(28.8)		
No	29	(21.2)	42	(71.2)		

^1^ N = 196.

**Table 5:** Reasons associated with reporting behavior regarding suspected concussion symptoms according to generalized linear model.

Question	Adjusted GLM				Unadjusted GLM			
	Wald	OR	(95% CI)	*p*	Wald	OR	(95% CI)	*p*
I realized it was an injury/symptom that I should report. (Neither)	Reference				Reference			
Negative	3.05	0.24	(0.05–1.19)	0.081	5.17	0.29	(0.10–0.84)	0.023
Positive	15.71	14.25	(3.83–53.03)	<0.001	16.75	5.57	(2.46–12.67)	<0.001
I did not realize at the time that I suffered a suspected concussion. (Neither)	Reference				Reference			
Negative	8.40	0.12	(0.03–0.50)	0.004	4.53	0.42	(0.19–0.93)	0.033
Positive	15.68	26.30	(5.21–132.67)	<0.001	11.90	4.86	(1.98–11.92)	0.001
I wanted to continue with the game/practice that day. (Neither)	Reference				Reference			
Negative	6.91	0.11	(0.02–0.57)	0.009	10.61	0.26	(0.12–0.59)	0.001
Positive	5.10	6.22	(1.27–30.40)	0.024	11.97	5.58	(2.11–14.77)	0.001
I thought that reporting the symptom might ruin the atmosphere of the team at games/practices. (Neither)	Reference				Reference			
Negative	6.39	6.68	(1.53–29.16)	0.011	0.55	1.36	(0.60–3.04)	0.459
Positive	0.01	1.05	(0.32–3.47)	0.940	1.14	1.50	(0.71–3.16)	0.285
I realized that I had symptoms of a suspected concussion (Neither)	Reference				Reference			
Negative	1.13	0.36	(0.06–2.35)	0.288	10.67	0.18	(0.06–0.50)	0.001
Positive	5.73	3.85	(1.28–11.59)	0.017	5.64	2.64	(1.18–5.89)	0.018
I thought others would think less of me if I reported my symptoms. (Neither)	Reference				Reference			
Negative	1.47	3.09	(0.50–19.06)	0.225	10.12	6.80	(2.09–22.15)	0.001
Positive	7.51	0.11	(0.02–0.54)	0.006	0.12	0.88	(0.43–1.81)	0.728
There was a doctor or trainer. (Neither)	Reference				Reference			
Negative	9.80	17.11	(2.89–101.29)	0.002	1.53	1.68	(0.74–3.80)	0.216
Positive	0.71	1.79	(0.46–6.94)	0.400	12.58	3.92	(1.84–8.35)	<0.001
I wanted to continue with the game/practice at next day. (Neither)	Reference				Reference			
Negative	7.14	0.09	(0.02–0.52)	0.008	0.05	0.91	(0.40–2.07)	0.820
Positive	2.15	3.41	(0.66–17.60)	0.142	17.51	5.84	(2.56–13.36)	<0.001
There was no doctor or trainer. (Neither)	Reference				Reference			
Negative	10.46	26.47	(3.64–192.72)	0.001	1.74	1.86	(0.74–4.68)	0.187
Positive	0.04	0.86	(0.20–3.65)	0.837	1.11	1.47	(0.72–3.02)	0.292
I thought that leaving game/practice would affect my future athletic career. (Neither)	Reference				Reference			
Negative	6.39	11.35	(1.73–74.60)	0.011	0.71	1.45	(0.61–3.40)	0.399
Positive	0.73	0.49	(0.09–2.54)	0.393	3.39	2.04	(0.95–4.36)	0.066
I felt pressure from others to report my symptoms. (Neither)	Reference				Reference			
Negative	0.12	1.26	(0.34–4.68)	0.727	1.72	1.82	(0.74–4.46)	0.190
Positive	1.20	0.42	(0.09–1.98)	0.273	0.00	1.02	(0.46–2.26)	0.954

Note: GLM, generalized linear model; OR, odds ratio.

## Data Availability

The data presented in this study are available upon request from the corresponding author.

## Supplementary Materials

The following supporting information can be downloaded at: https://www.mdpi.com/article/10.3390/ijerph20032569/s1, Table S1: Player profile; Table S2: Concussion education; Table S3: Number of experiences with suspected concussion symptoms; Table S4: Experience of suspected concussion symptoms and subsequent behavior.

## Appendix A

Q1. Please answer the following questions about your profile using numbers.

Age:     years old:     Grade:      Playing year:    years

Q2. From the list below, select if you have any of the symptoms of a suspected concussion.

	**Signs/Symptoms**		
①	Headache	Applicable	Not applicable
②	“Pressure in head”	Applicable	Not applicable
③	Neck pain	Applicable	Not applicable
④	Neck muscle weakness	Applicable	Not applicable
⑤	Joint stiffness	Applicable	Not applicable
⑥	Numbness in arms	Applicable	Not applicable
⑦	Nausea or vomiting	Applicable	Not applicable
⑧	Dizziness	Applicable	Not applicable
⑨	Blurred vision	Applicable	Not applicable
⑩	Black eye	Applicable	Not applicable
⑪	Bleeding from the ears	Applicable	Not applicable
⑫	Bleeding from the mouth	Applicable	Not applicable
⑬	Nosebleed	Applicable	Not applicable
⑭	Balance problems	Applicable	Not applicable
⑮	Sensitivity of light	Applicable	Not applicable
⑯	Sensitivity of noise	Applicable	Not applicable
⑰	Abnormal sense of smell	Applicable	Not applicable
⑱	Abnormal sense of taste	Applicable	Not applicable
⑲	Feeling slowed down	Applicable	Not applicable
⑳	Feeling like “in a fog”	Applicable	Not applicable
㉑	“Don’t feel right”	Applicable	Not applicable
㉒	Difficulty concentrating	Applicable	Not applicable
㉓	Difficulty remembering	Applicable	Not applicable
㉔	Fatigue or low energy	Applicable	Not applicable
㉕	Fever	Applicable	Not applicable
㉖	Skin rash	Applicable	Not applicable
㉗	Confusion	Applicable	Not applicable
㉘	Drowsiness	Applicable	Not applicable
㉙	More emotional	Applicable	Not applicable
㉚	Irritability	Applicable	Not applicable
㉛	Sadness	Applicable	Not applicable
㉜	Nervous or anxious	Applicable	Not applicable
㉝	Trouble falling asleep	Applicable	Not applicable

Q3. Have you ever been educated on the symptoms of a suspected concussion, what to do after a concussion occurs, and procedures for returning to play?

 □Yes (Please go to Q4.)

 □No (Please go to Q6.)

Q4. When did you receive education about concussions? Please check all that apply.

 □Elementary school students  □Junior high school students 

 □High school students  □Collegiate students

Q5. What form of education about concussions did you receive? Please check all that apply.

 □School class □Instruction by club advisor □Instruction by trainer □Books

 □Websites □SNS □Video sites (ex. YouTube) □Others (         )

Q6. Have you experienced any of the symptoms listed below during rugby when (1) you hit your head or (2) your head was shaken, although there was no direct impact on your head?

	**Signs/Symptoms**		
①	Headache	Experienced	No experienced
②	“Pressure in head”	Experienced	No experienced
③	Neck pain	Experienced	No experienced
④	Nausea or vomiting	Experienced	No experienced
⑤	Dizziness	Experienced	No experienced
⑥	Blurred vision	Experienced	No experienced
⑦	Balance problems	Experienced	No experienced
⑧	Sensitivity to light	Experienced	No experienced
⑨	Sensitivity to noise	Experienced	No experienced
⑩	Feeling slowed down	Experienced	No experienced
⑪	Feeling like “in a fog”	Experienced	No experienced
⑫	“Don’t feel right”	Experienced	No experienced
⑬	Difficulty concentrating	Experienced	No experienced
⑭	Difficulty remembering	Experienced	No experienced
⑮	Fatigue or low energy	Experienced	No experienced
⑯	Confusion	Experienced	No experienced
⑰	Drowsiness	Experienced	No experienced
⑱	More emotional	Experienced	No experienced
⑲	Irritability	Experienced	No experienced
⑳	Sadness	Experienced	No experienced
㉑	Nervous or anxious	Experienced	No experienced
㉒	Trouble falling asleep	Experienced	No experienced

If you did not select any of the “Experienced”, your answer is complete.

Thank you for your participation.

If you selected at least one of the following, please go to Q7.

Q7. How many times have you experienced the above symptoms?

Total      times

Please provide a breakdown.

Elementary school students:      times

Junior high school students:      times

High school students:      times

Collegiate students:      times

Q8. When was the most recent symptom you experienced so far?

 □Immediately after hitting your head □After practice/games □On the way home

 □From arriving home to having a meal □When taking a bath

 □From leaving the bath to going to bed □In the middle of sleeping

 □When waking up the next morning □After that

Q9. Have you reported your latest symptoms to anyone?

 □Yes □No

For the following questions, please answer the last time you experienced any of the symptoms listed above.

Q10. Have you received medical attention by a doctor after the symptoms described above?

 □Yes □No

Q11. Which situation did you experience the symptoms described above?

 □Match □Practice □Other activity

Q12. When you experienced any of the symptoms listed above, did people around you identify the symptoms?

 □Yes

 □No

Q13. When you experienced the symptoms described above, did you report them yourself to the people around you?

 □Yes (Please go to Q14.)

 □No (Please go to Q16)

Q14. To whom did you report the symptoms you felt after the impact on your head? Please check all that apply.

 □Club advisor/coach □Trainer □Doctor □Teammates □Parents □Others

Q15. When you reported the symptoms described above to someone to anyone, how much did you agree with each of the following questions?

		Not think← →Think
①	I reported it because I realized it was an injury/symptom that I should report.	1 2 3 4 5
②	I didn’t realize at the time that I suffered a suspected concussion, but I thought it was a symptom I should report.	1 2 3 4 5
③	I wanted to continue with the match/practice that day, but I thought it was a symptom I should report.	1 2 3 4 5
④	I thought that reporting the symptom might ruin the team atmosphere at the match/practice, but I thought it was a symptom that should be reported.	1 2 3 4 5
⑤	I realized that symptoms of a suspected concussion.	1 2 3 4 5
⑥	I thought others would think less of me if I reported my symptoms.	1 2 3 4 5
⑦	There was a doctor or trainer, and I reported symptoms.	1 2 3 4 5
⑧	I wanted to continue with match/practice at next day, but I thought it was a symptom that should be reported.	1 2 3 4 5
⑨	There was no doctor or trainer, but I reported symptoms.	1 2 3 4 5
⑩	I thought that leaving match/practice would affect my future athletic career, but I thought it was a symptom that should be reported.	1 2 3 4 5
⑪	I felt pressure from others to report my symptoms, but I thought I should report them.	1 2 3 4 5

Q16. When you did not report any of the above symptoms to anyone, how much did you agree with each of the following questions?

		Not think← →Think
①	I didn’t report the injury/symptom, even though I realized it was an injury/symptom that I should report.	1 2 3 4 5
②	I didn’t report symptoms at the time because I didn’t realize at the time that I suffered a suspected concussion.	1 2 3 4 5
③	I didn’t report symptoms because I wanted to continue with the match/practice that day.	1 2 3 4 5
④	I didn’t report symptoms because I thought that reporting them might ruin the team atmosphere at the match/practice.	1 2 3 4 5
⑤	I didn’t report symptoms but I realized that symptoms of a suspected concussion.	1 2 3 4 5
⑥	I didn’t report symptoms because I thought others would think less of me if I reported them.	1 2 3 4 5
⑦	I didn’t report symptoms but there was a doctor or trainer.	1 2 3 4 5
⑧	I didn’t report symptoms because I wanted to continue with match/practice at next day.	1 2 3 4 5
⑨	I didn’t report symptoms because there was no doctor or trainer.	1 2 3 4 5
⑩	I didn’t report symptoms because I thought that leaving match/practice would affect my future athletic career.	1 2 3 4 5
⑪	I didn’t report symptoms because I felt pressure from others to report them.	1 2 3 4 5
