# Supplementary material for: Reasons for the Reporting Behavior of Japanese Collegiate Rugby Union Players Regarding Suspected Concussion Symptoms: A Propensity Analysis
Source: Int J Environ Res Public Health. 2023 Jan 31;20(3):2569. doi: 10.3390/ijerph20032569 (PMC9915167; doi:10.3390/ijerph20032569)
Supplement: Supplementary file 1 [file ijerph-20-02569-s001.zip › Supplementary Table S1.pdf]

Table S1. Player profile

Demographic	N = 208	
Age (year)	20.3	(20.1–20.5)
No. of years played	9.1	(8.6–9.7)
Grade in college		
Freshman	52	(25.0)
Sophomore	51	(24.5)
Junior	53	(25.5)
Senior	52	(25.0)

Continuous data are presented as mean and 95% CI. Categorical data are presented as frequency and percentage (%).
