# Supplementary material for: Reasons for the Reporting Behavior of Japanese Collegiate Rugby Union Players Regarding Suspected Concussion Symptoms: A Propensity Analysis
Source: Int J Environ Res Public Health. 2023 Jan 31;20(3):2569. doi: 10.3390/ijerph20032569 (PMC9915167; doi:10.3390/ijerph20032569)
Supplement: Supplementary file 1 [file ijerph-20-02569-s001.zip › Supplementary Table S2.pdf]

Table S2. Experiences of suspected concussion symptoms in collegiate rugby union players

Questions	Frequency	(%)
Have you ever been educated on the symptoms of a suspected concussion, what to do after a concussion occurs, and procedures for returning to play?[1]		
Yes	121	(58.2)
No	87	(41.8)
When did you receive education about concussions?[2]		
Elementary school students	7	(3.7)
Junior high school students	28	(14.9)
High school students	94	(50.0)
Collegiate students	59	(31.4)
What form of education about concussions did you receive? (Multiple answers were acceptable.)[3]		
School class	45	(20.7)
Instruction by club advisor	87	(40.1)
Instruction by trainer	60	(27.6)
Books	2	(0.9)
Websites	9	(4.1)
SNS	7	(3.2)
Video sites (ex. YouTube)	4	(1.8)
Others	3	(1.4)

[1] N = 208. [2] N = 188. [3] N = 217.
