# Supplementary material for: Reasons for the Reporting Behavior of Japanese Collegiate Rugby Union Players Regarding Suspected Concussion Symptoms: A Propensity Analysis
Source: Int J Environ Res Public Health. 2023 Jan 31;20(3):2569. doi: 10.3390/ijerph20032569 (PMC9915167; doi:10.3390/ijerph20032569)
Supplement: Supplementary file 1 [file ijerph-20-02569-s001.zip › Supplementary Table S3.pdf]

Table S3. Number of experiences with suspected concussion symptoms

Questions	Mean	(95% CI)
How many times have you experienced the above symptoms?		
Elementary school students	0.1	(0.05–0.14)
Junior high school students	0.3	(0.23–0.40)
High school students	1.6	(1.32–1.78)
Collegiate students	0.9	(0.70–1.03)
