# Supplementary material for: Reasons for the Reporting Behavior of Japanese Collegiate Rugby Union Players Regarding Suspected Concussion Symptoms: A Propensity Analysis
Source: Int J Environ Res Public Health. 2023 Jan 31;20(3):2569. doi: 10.3390/ijerph20032569 (PMC9915167; doi:10.3390/ijerph20032569)
Supplement: Supplementary file 1 [file ijerph-20-02569-s001.zip › Supplementary Table S4.pdf]

Table S4. Experience of suspected concussion symptoms and subsequent behavior

Questions	Frequency	(%)
Have you received medical attention by a doctor after experiencing the suspected concussion symptoms?[1]		
Yes	60	(30.6)
No	135	(68.9)
Not answered	1	(0.5)
Which situation did you experience the suspected concussion symptoms?[1]		
Match	145	(74.0)
Practice	51	(26.0)
Other activity	0	(0.0)
Did people around you detect the symptoms?[1]		
Yes	125	(63.8)
No	71	(36.2)
Did you report symptoms yourself to the people round you?[1]		
Yes	137	(69.9)
No	59	(30.1)
To whom did you report the symptoms you felt after the impact on your head? (Multiple answers were acceptable.)[2]		
Club advisor / coach	104	(31.3)
Trainer	77	(23.2)
Doctor	34	(10.2)
Teammates	64	(19.3)
Parents	52	(15.7)
Others	1	(0.3)

[1] N = 196. [2] N = 332.
